# Naldemedine for the management of opioid-induced constipation in patients with cancer pain: A narrative review

**DOI:** 10.1097/MD.0000000000043644

**Published:** 2025-08-01

**Authors:** Hiromi Nishiba, Hisao Imai, Ayumu Nagamine, Yuta Takahashi, Kyoko Obayashi

**Affiliations:** a Graduate School of Pharmaceutical Sciences, Takasaki University of Health and Welfare, Takasaki, Japan; b Division of Pharmacy, Japan Community Health Care Organization (JCHO) Gunma Chuo Hospital, Maebashi, Japan; c Department of Respiratory Medicine, Comprehensive Cancer Center, International Medical Center, Saitama Medical University, Hidaka-City, Japan; d Education Center for Clinical Pharmacy, Faculty of Pharmacy, Takasaki University of Health and Welfare, Takasaki, Japan.

**Keywords:** analgesia, cancer pain, naldemedine, opiates, opioid-induced bowel dysfunction, opioid-induced constipation, treatment

## Abstract

The prevalence of chronic pain in patients with cancer ranges from 23% to 36%. Opioids are effective in relieving severe pain; however, they also cause side effects, such as physical dependence, tolerance, sedation, and hyperalgesia. The prevalence of opioid-induced constipation (OIC) increases with a rise in the duration of opioid analgesic use. When OIC develops, opioid treatment satisfaction decreases, and many patients tend to discontinue treatment. Naldemedine is an opioid receptor antagonist that improves OIC in patients with cancer by acting on the gastrointestinal tract. It does not inhibit opioid analgesia or cause withdrawal symptoms. Therefore, this review aims to provide a comprehensive overview of OIC management in patients with cancer pain, with a focus on naldemedine. This narrative review also reports recent findings on the efficacy and safety of naldemedine and provides comparisons based on cancer type. A comprehensive search was conducted to identify primary articles on the clinical effectiveness and adverse effects of naldemedine in patients with cancer with OIC. Naldemedine is a well-tolerated and effective drug for treatment of OIC in patients with cancer, and its use is expected to increase.

## 1. Introduction

Reports indicate that the prevalence of chronic pain in patients with cancer ranges between 22.5% and 35.4%.^[1]^ A systematic review of chronic pain among patients with cancer over the past 40 years reported a prevalence of 40% to 74%.^[2]^ Because of the intensity and frequency of chronic pain in patients with cancer, a higher prevalence of depression and poor quality of life have also been observed.^[3]^ Opioids are more effective than non-opioid analgesics in controlling moderate-to-severe pain. Their primary mechanism involves binding to opioid receptors densely located within the brain and central nervous system, thereby attenuating the transmission of nociceptive signals and altering the subjective experience of pain without addressing its underlying cause.^[4]^ In addition to their central actions, opioids exert peripheral effects through receptor activation in various organ systems – including smooth muscle, the adrenal glands, myocardium, vascular endothelium, gastrointestinal tract, and pulmonary tissue. These interactions are associated with a broad spectrum of physiological effects, such as respiratory depression, modulation of fluid and hormonal homeostasis, reduced gastrointestinal motility, urinary retention, and sedation.^[5]^ Although opioids are effective in alleviating severe pain, they also cause adverse effects, such as physical dependence, tolerance, sedation, and hyperalgesia.^[6]^ Opioid-induced constipation (OIC) is a persistent and significant condition that exhibits varying use and response to laxatives, thereby exacerbating the ongoing burden of chronic pain.^[7]^ Opioid treatment satisfaction declines when OIC develops, leading many patients to reduce their opioid therapy once constipation occurs.^[8]^ Consequently, addressing OIC is crucial for maintaining effective opioid treatment. Typically, OIC is treated with laxatives; however, newer pharmacological interventions, such as peripherally acting mu-opioid receptor antagonists (PAMORAs), have been introduced. Among these, naldemedine is the most recent opioid receptor antagonist frequently used in clinical practice. Therefore, this review aims to present the current state of knowledge regarding the management of OIC in patients with cancer pain. Notably, the majority of research on naldemedine originates from Japan, where naldemedine is the sole PAMORA available on the market. Therefore, this review focuses on naldemedine.

## 2. OIC

Gastrointestinal adverse effects have a major impact on the health and quality of life of opioid users, with OIC being the most common gastrointestinal adverse effect.^[8]^ OIC results in changes in defecation patterns and habits and may coincide with defecation with overflow incontinence and Opioid-induced bowel dysfunction (OIBD), such as reflux, nausea, and bloating, in some patients. Additionally, OIC, which is characterized by functional constipation, is defined as a change in defecation frequency or bowel habits or patterns after the initiation of opioid therapy.^[9]^ Opioid analgesics are regarded as the primary class of medication among those that constrain bowel movements in patients with cancer.^[10]^ Constipation is the most prevalent adverse effect of all opioids, affecting 59% of patients with cancer receiving opioid analgesics.^[11]^ Its prevalence varies depending on the specific opioid type and administration route.

Table 1 presents the Rome IV criteria for OIC.^[9]^ A recent multicenter observational study, conducted in real-world clinical settings and involving 1000 patients, revealed that the Rome IV diagnostic criteria for OIC demonstrated high sensitivity (85%) and specificity (77%), making it a reliable diagnostic tool.^[11]^ Furthermore, when diagnosed using the international Rome IV diagnostic criteria for OIC, the incidence of OIC was reported to be 56% in a survey conducted in Japan.^[12]^

**Table 1:** Rome IV criteria for opioid-induced constipation.

1. New or worsening symptoms of constipation when initiating, changing, or increasing opioid therapy that must include 2 or more of the following:
i. Straining during > 25% of defecations
ii. Lumpy or hard stools (BSFS 1–2) in > 25% of defecations
iii. Sensation of incomplete evacuation during > 25% of defecations
iv. Sensation of anorectal obstruction/blockage during > 25% of defecations
v. Manual maneuvers to facilitate > 25% of defecations (e.g., digital evacuation or support of the pelvic floor)
vi. Fewer than 3 SBM per week
2. Loose stools are rarely present without the use of laxatives

BSFS = Bristol stool form scale, SBM = spontaneous bowel movement.

In recent decades, numerous small-scale studies have reported a higher incidence of OIC associated with hydrophilic opioids – such as morphine, oxycodone, and hydromorphone – than with lipophilic opioids such as methadone, fentanyl, buprenorphine, and tapentadol. This trend is particularly evident when opioids are administered orally.^[13]^ A large retrospective cohort study found no statistically significant differences between transdermal therapeutic systems and orally administered opioids.^[14]^ However, opioids constitute only one of many contributing factors to constipation, with comorbidities, clinical conditions, medications, as well as dietary and behavioral influences playing a crucial role in its development. A recently published Cochrane review found no distinct differences in constipation frequency among hydromorphone, oxycodone, morphine, and fentanyl.^[15]^

Among the various gastrointestinal side effects of opioids, constipation is one of the most prevalent. OIBD is a pharmacologically mediated syndrome characterized by a constellation of gastrointestinal symptoms. These may include xerostomia, gastroesophageal reflux, nausea, vomiting, abdominal bloating and discomfort, anorexia, hard or infrequent stools, constipation, and a persistent sensation of incomplete evacuation^[16]^ The pathogenesis of OIC is driven by the interaction of opioids with receptors located throughout the gastrointestinal tract, leading to altered motility, secretion, and coordination of bowel function.^[16]^ The gastrointestinal tract expresses all 3 major classes of opioid receptors – delta (δ), kappa (κ), and mu (μ) – each contributing to the modulation of enteric neural and smooth muscle activity.^[17]^ In humans, μ-opioid receptors play a predominant role in the pathophysiology of OIBD. These receptors are localized primarily to submucosal and myenteric neurons, as well as to mononuclear cells within the lamina propria.^[17]^ Whereas endogenous opioid peptides modulate physiological gastrointestinal function under normal conditions, exogenous opioids similarly engage these receptors, often leading to dysregulation of motility and secretion.^[18]^ All opioid receptors are members of the G protein–coupled receptor superfamily and mediate intracellular signaling through multiple mechanisms. These include activation of potassium (K⁺) channels, leading to membrane hyperpolarization, and inhibition of calcium (Ca²⁺) channels, resulting in reduced neurotransmitter release. Additionally, modulation of sodium (Na⁺) channels may also contribute to their downstream effects.^[19]^ However, the principal mechanism is thought to involve the suppression of cyclic adenosine monophosphate synthesis, which in turn modulates downstream effector pathways, ultimately reducing neuronal excitability and exerting an overall inhibitory effect on target cells.^[20]^ The effects of opioid agonism on the gastrointestinal tract can be summarized as follows: (i) a reduction in propulsive peristalsis, resulting in delayed intestinal transit; (ii) increased sphincter constriction and diminished rectal wall reflex; and (iii) decreased secretion of water and electrolytes in the small intestine.^[21]^ Additionally, delayed intestinal transit enhances water reabsorption, leading to the formation of hard, lumpy stools.

OIC imposes a significant burden on patients, adversely impacting their health-related quality of life.^[22]^ Consequently, it becomes an increasingly important concern when opioids are administered to terminally ill patients, who may exhibit poor tolerance to increased gastrointestinal side effects.^[23]^ The well-being of patients with OIC is influenced by a multitude of factors, including symptom severity, disruptions in daily and occupational activities, emotional distress such as frustration, anxiety, and depression, feelings of helplessness and disgust, as well as concerns related to privacy. The onset of chronic constipation associated with prolonged opioid therapy can lead to significant and potentially long-lasting clinical consequences, such as colonic distension, ileus, and perforation.^[22]^ Moreover, OIC complicates opioid therapy, as patients strive to balance effective pain management with the risk of constipation, often leading to adjustments or discontinuation of treatment.^[24]^

Incremental costs associated with treatment include direct and indirect costs such as medication; home, outpatient clinic, and emergency outpatient visits; and testing costs.^[22]^ Patients with cancer and OIC face twice the risk of inpatient or pain-related hospitalization compared to those without constipation.^[25]^ Specifically, the overall annual cost of treatment increases by 36% and pain-related costs by 72%.^[25]^ Conventional OIC treatment includes non-pharmacological methods, such as the intake of a diet high in dietary fiber, and the use of drugs such as laxatives. Other osmotic laxatives such as polyethylene glycol are also used in many countries to treat and prevent OIC. However, neither dietary therapy nor osmotic laxative treatment targets the pathogenic mechanism of OIC.^[26–28]^ Methylnaltrexone, naldemedine, and naloxegol are pharmacological agents that block μ-opioid receptors in the gastrointestinal tract, thereby reducing constipation associated with opioid therapy without affecting the central analgesic effects of opioids.^[29,30]^ Naldemedine and naloxegol, which are oral medications administered once daily, have demonstrated efficacy in the treatment of OIC in patients with cancer, improving quality of life while producing relatively mild side effects.^[31,32]^ Methylnaltrexone can be administered in 2 forms: once-daily tablets and subcutaneous injections. Subcutaneous methylnaltrexone is the sole PAMORA available for OIC treatment in adults with advanced disease or active cancer-induced pain. All 3 PAMORAs are used for the treatment of adult patients with cancer presenting with OIC.^[30,32]^ Table 2 lists the characteristics of the PAMORAs.^[33–36]^

**Table 2:** Comparison of peripherally acting mu-opioid receptor antagonists.

Category	PAMORAs^[33]^		
	Methylnaltrexone^[34,35]^	Naloxegol^[36]^	Naldemedine
Features	The only PAMORA that can be injected subcutaneously	Pegylated derivative of the μ-opioid receptor antagonist naloxone	σ-opioid receptor antagonist that suppresses nausea and vomiting^[37,38]^
Mechanism	Peripherally acting μ-receptor antagonist	The polyethylene glycol portion limits the ability of naloxegol to cross the blood–brain barrier	Peripheral μ, σ, and κ opioid receptor antagonists
Route of administration	Oral; subcutaneous	Oral	Oral
Metabolism	Conversion to methyl-6-naltrexol isomers and methylnaltrexone sulfate	Mainly CYP3A4	Mainly CYP3A4
Side effects	Abdominal pain Flatulence Nausea Dizziness	Abdominal pain Diarrhea Nausea Flatulence	Abdominal pain Diarrhea Nausea Gastroenteritis

CYP3A4 = cytochrome P450 family 3 subfamily A member 4, PAMORA = peripherally acting mu-opioid receptor antagonist.

## 3. Naldemedine

The generic name of naldemedine is naldemedinetosylate. In a phase-IIb trial, participants were randomized to receive 0.1, 0.2, or 0.4 mg of naldemedine or placebo. The proportion of spontaneous bowel movement (SBM) responders, defined as patients with ≥ 3 SBMs/week and an increase of ≥ 1 SBM/week from baseline over the last 2 weeks of treatment, was significantly higher after administration of 0.2 and 0.4 mg of naldemedine, but not 0.1 mg. No significant differences were found between a dose of 0.2 and 0.4 mg of naldemedine. The incidence of adverse events increased with increasing doses of naldemedine. Based on these results, a dose of 0.2-mg naldemedine was concluded to be the optimal dose with the best benefit and risk profile.^[39]^ For adults, naldemedine is administered orally at a dose of 0.2 mg once daily.^[40]^ Naldemedine is a noncompetitive antagonist that does not require dose adjustment.^[41]^

### 3.1. Mechanism of action

Naldemedine is a drug that acts as a peripheral antagonist of μ-, δ-, and κ-opioid receptors. It alleviates OIC by binding to peripheral opioid receptors without affecting central opioid receptors. This is due to its large polar side chains, which prevent it from crossing the blood–brain barrier.^[42]^ Peripheral δ- and κ-opioid receptors are primarily located in the proximal stomach and colon, while μ receptors are widely distributed throughout the gastrointestinal tract.^[18,43]^ Therefore, μ-opioid receptors in the enteric nervous system act peripherally on OIC to improve symptoms.^[44]^

### 3.2. Pharmacokinetics/pharmacodynamics

Following oral administration, naldemedine reaches its peak concentration (C_max_) within 0.5 to 3.0 hours (T_max_). In a fasted state, peak concentrations are achieved after a median of 0.75 hours. A high-fat meal delays T_max_ to 2.5 hours and reduces C_max_ by approximately 35% without any significant impact on overall absorption (as indicated by the area under the curve). The area under the plasma concentration–time curve and C_max_ are dose-dependent and almost dose-proportional, with minimal accumulation following multiple daily doses. Naldemedine is 93% to 94% bound to plasma proteins and has a mean apparent volume of distribution of 155 L. It is primarily metabolized by hepatic cytochrome P450 3A (CYP3A) to nor-naldemedine and secondarily metabolized by uridine diphosphate glucuronosyltransferase 1A3 (UGT1A3) to naldemedine 3-glucuronide (3-G). Both metabolites are less potent antagonists than naldemedine. Naldemedine has a clearance rate of 8.4 L/h and a terminal elimination half-life of approximately 11 hours. Following a single oral dose, 57% of naldemedine is excreted in the urine, with 16% to 20% remaining unchanged and 35% excreted in the feces.^[45]^

Naldemedine undergoes primary metabolism via CYP3A4 to form nor-naldemedine, with some contribution from UGT1A3 to the formation of both naldemedine 3-G and 6-G. Neither naldemedine nor its major metabolite, nor-naldemedine, cause clinically significant induction or inhibition of major CYP450 enzymes. Additionally, naldemedine is a sensitive substrate of P-glycoprotein (P-gp), and no significant inhibition of major transporters has been observed.^[40]^

### 3.3. Hepatic and renal impairment

Naldemedine does not require dose adjustments in patients with mild, moderate, or severe renal impairment or those with end-stage renal disease (glomerular filtration rate < 30 mL/min/1.73 m^2^). Similarly, the pharmacokinetics in patients with mild or moderate hepatic impairment (Child–Pugh classes A and B) is comparable to that in healthy patients.^[46]^ However, data are unavailable for patients with severe liver failure, as opioids may worsen this clinical condition.^[46]^

### 3.4. Drug–drug interactions

Naldemedine, an amide derivative of the opioid receptor antagonist naltrexone, possesses a high molecular weight and extensive polar surface area and functions as a substrate of the P-gp efflux transporter.^[42]^ Naldemedine is predominantly metabolized by CYP3A4, yielding nor-naldemedine as its primary metabolite. Additionally, it serves as a substrate for P-gp and undergoes further metabolism via CYP3A. Co-administration with P-gp inhibitors, CYP3A inhibitors, or CYP3A inducers can significantly alter its pharmacokinetics and safety profile. Specifically, cyclosporine increases the C_max_ of naldemedine by 1.45-fold, AUC_0–last_ by 1.79-fold, and AUC_0–inf_ by 1.78-fold; itraconazole elevates C_max_ by 1.12-fold, AUC_0–last_ by 2.65-fold, and AUC_0–inf_ by 2.91-fold; and conversely, rifampin reduces C_max_ by 38%, AUC_0–last_ by 83%, and AUC_0–inf_ by 83% compared to naldemedine monotherapy.^[47,48]^ The concurrent administration of naldemedine with CYP3A4 inhibitors (such as ketoconazole, itraconazole, saquinavir, and clarithromycin) should be avoided, as it may lead to increased naldemedine levels and adverse effects. Conversely, the concurrent administration of strong CYP3A4 inducers (such as carbamazepine, phenytoin, and rifampicin) may result in decreased levels of naldemedine.^[47]^

## 4. Clinical data and trials of naldemedine in patients with cancer pain

Table 3 summarizes the results of studies on naldemedine, showing the results of prospective and retrospective studies.

**Table 3:** Summary of studies of naldemedine.

	Study	Number of patients	Efficacy*	Safety†
Prospective	Katakami et al 2017 Japan^[49]^	Effectiveness analysis sets (n = 225) Safety analysis sets (n = 226)	0.1 mg, 0.2 mg, 0.4 mg 56.4%, 77.6%, 82.1%	0.1 mg, 0.2 mg, 0.4 mg 66.1%, 67.2%, 78.6%
	Katakami et al 2017 Japan^[50]^ Compose-4	193	71.10%	44.30%
				(Diarrhea 19.6%, nausea 1.0%)
	Katakami et al 2017 Japan^[50]^ Compose-5	131	Not reported	80.20%
				(Diarrhea 18.3%, nausea 13.0%)
	Takata et al 2022 Japan^[51]^	Safety analysis sets (n = 1177) Effectiveness analysis sets (n = 953)	75%‡	11.3%§
				(Diarrhea 9.09%, nausea 0.25%)
	Shimizu et al 2024 Japan^[52]^	184	62.70%	Diarrhea 17.6%
	Ozaki et al 2022 Japan^[53]^	120	The Japanese Patient Assessment of Constipation Quality of Life and complete spontaneous bowel movement *P < *.001 MaO 0.4, Nal 0.03	27% (Diarrhea 10.0%)
	Hamano et al 2024 Japan^[37]^	99	64.6%**	29%
Retrospective	Fujita et al 2023 Japan^[54]^	71	66.10%	Diarrhea 38.0%
	Takagi et al 2020 Japan^[55]^	98	Daily defecation counts increased after late (median 0.43–0.88, *P* < .001) but remained stable after the early naldemedine administration (median 1.00–1.00, *P* = .34)‖	20% (Diarrhea 17%; earlier than in the late group [3.9% vs 22.2%])¶
	Hiruta et al 2020 Japan^[56]^	296	79.60%	Diarrhea 29.4% (grade 1, 20.6%)
				Nausea 9.5% (grade 1, 5.7%)#
	Nishiba et al 2022 Japan^[57]^	149	65.70%	Diarrhea 38.2% (grade 1, 88.1%)
				Nausea 9.4% (grade 1, 6.0%)#

AE = adverse event, PS = performance status.

* Proportion of spontaneous bowel movement responders.

† Treatment-emergent AEs (AE version 4.0).

‡ Effectiveness of treatment based on improvement in the frequency of bowel movements (improved, unchanged, or worsened).

§ Medical dictionary for regulatory activities version 23.0.

‖ The Japanese Patient Assessment of Constipation Quality of Life score; lower scores indicate greater quality of life.

¶ Treatment-related AEs.

# Treatment-emergent AEs (AE version 5.0).

** Normal Bowel Function Index (BFI); the proportion of patients with a BFI of <28.8 on day 14.

### 4.1. Efficacy: prospective studies

#### 4.1.1. Phase-I trial

A phase-I study by Fukuhara et al assessed the safety, tolerability, and pharmacokinetic profile of oral naldemedine in healthy volunteers.^[58]^ It details 2 randomized, placebo-controlled, double-blinded phase-I trials, specifically, single ascending dose (SAD) and multiple ascending dose (MAD) studies. In the SAD study, 42 participants received a single dose of naldemedine (0.1–100 mg), while 14 received a placebo.^[58]^ In the MAD study, 27 participants received naldemedine (3–30 mg) or placebo (n = 9) once daily for 10 days.^[58]^ On day 1 of the SAD study and day 10 of the MAD study, the maximum plasma concentrations of naldemedine ranged from 1.98 to 2510 ng/mL and 73.8 to 700 ng/mL, respectively. The peak concentrations were observed at 0.5 and 0.75 hours, with urinary excretion ranging from 15.9% to 20.5% and 19.7% to 19.1%, respectively. No major safety or tolerability concerns were observed at doses that were 150 to 500 times the therapeutic dose of naldemedine (0.2 mg). The incidence of adverse events was dose-independent. Naldemedine was associated with a higher incidence of gastrointestinal adverse events than placebo, all of which were considered treatment-related. The drug was rapidly absorbed, and no safety or tolerability issues were observed at the doses assessed.^[58]^

#### 4.1.2. Phase-II trial

A randomized, double-blinded, multicenter study by Katakami et al compared the efficacy and safety of 3 doses of naldemedine with placebo in patients with cancer and OIC to determine the dose for future studies of the PAMORA naldemedine. Of the 227 randomized patients, 225 and 226 underwent efficacy (0.1 mg of naldemedine, n = 55; 0.2 mg, n = 58; 0.4 mg, n = 56; and placebo, n = 56) and safety assessments, respectively. The primary endpoint, which was changes in SBM frequency, showed higher values with all doses of naldemedine than with placebo (*P < *.05 for all comparisons). The number of SBM responders was higher in the naldemedine group than that in the placebo group. Additionally, the primary endpoint showed significant improvement (*P < *.05) with doses of 0.2 and 0.4 mg (but not 0.1 mg) of naldemedine compared with placebo. Specifically, naldemedine demonstrated a statistically significant improvement in the change in SBM frequency compared to placebo in the full analysis set (all patients randomized, received study treatment, and had efficacy data) over the 14-day treatment period (least-squares mean change from baseline and *P*-value: naldemedine .1 mg: 3.43, *P* = .0465; naldemedine 0.2 mg: 4.75, *P* < .001; naldemedine 0.4 mg: 7.29, *P* < .001; and placebo: 1.50). The naldemedine group experienced more adverse events during treatment (0.1 mg of naldemedine: 66.1%, 0.2 mg: 67.2%, and 0.4 mg: 78.6%) than the placebo group (51.8%). Diarrhea was the most common adverse event during treatment. Therefore, the recommended dose is 0.2 mg.^[49]^

#### 4.1.3. Phase-III trial

Katakami et al conducted a phase-III trial consisting of a 2-week, randomized, double-blinded, placebo-controlled trial (COMPOSE-4). Eligible patients had an Eastern Cooperative Oncology Group performance status (ECOG-PS) ≤ 2, any cancer type that did not directly affect the gastrointestinal function, and a cancer condition expected to remain stable for the duration of the study. A total of 290 patients were assessed for eligibility, and 193 were randomly assigned on a 1:1 basis to receive naldemedine (n = 97) or placebo (n = 96). The proportion of SBM responders during the 2-week treatment period of COMPOSE-4 (primary endpoint) was significantly greater with naldemedine (71.1% [69 of 97 patients]; 95% confidence interval [CI], 61.0–79.9%) than with placebo (34.4% [33 of 96 patients]; 95% CI, 25.0–44.8%), with a difference of 36.8% (95% CI, 23.7–49.9%; *P < *.0001). A significantly greater change from the baseline was observed with naldemedine than with placebo in the least-squares mean frequency of SBMs/week (5.16 vs 1.54; a difference of 3.62 [95% CI, 2.13–5.12; *P < *.0001]); complete SBMs/week (2.76 vs 0.71; a difference of 2.05 [95% CI, 1.29–2.81; *P < *.0001]); and SBMs without straining/week (3.85 vs 1.17; a difference of 2.67 [95% CI, 1.20–4.15; *P = *.0005]).^[50]^ The primary objective of COMPOSE-4 (proportion of SBM responders) was achieved. Additionally, the efficacy of naldemedine vs placebo was also demonstrated by significant increases in the frequency of SBMs/week, complete SBMs/week, and SBMs without straining/week. Naldemedine was generally well-tolerated in this study population, even in those patients who received a daily dose for 14 weeks. Furthermore, naldemedine was associated with a higher incidence of treatment-emergent adverse events (TEAEs) than placebo; however, most of these TEAEs were mild to moderate in severity and led to study discontinuations in only a few patients (9.3%). Naldemedine was generally well-tolerated in patients with OIC and concurrent cancer.^[50]^

### 4.2. Efficacy: post-marketing study

A sub-analysis of post-marketing surveillance investigated treatment-related side effects of long-term 12-week treatment in 1182 patients. In this study, naldemedine (0.2 mg, once daily) was orally administered for up to 12 weeks. In the efficacy analysis population (n = 953), both the frequency of bowel movements (75.0% and 83.2%) and voiding status (80.0% and 88.0%) improved at weeks 2 and 12, respectively. Patients who were excluded from clinical trials (e.g., ECOG-PS ≥ 3) or who constituted a relatively small proportion of the study population (e.g., those receiving weak opioids) also demonstrated improvements in bowel movement frequency and bowel status. The incidence of treatment-related side effects within subgroups related to age, presence of brain metastases, opioids used at the initiation of naldemedine, and concomitant laxatives did not significantly differ.^[51,59]^

### 4.3. Efficacy: retrospective and prospective observational studies

A total of 149 patients were included in a real-world retrospective study, of whom 117 died.^[57]^ The mean time from the commencement of naldemedine treatment to death was 35 (range, 7–790) days. Of the 149 patients included in the analysis, 89 were men, with a median age of 72 (range, 38–96) years. Additionally, 60 (40.2%) patients were > 75 years of age. According to the ECOG criteria, 40 (26.8%) patients had a PS of 0 to 1, 38 (25.5%) had a PS of 2, and 71 (47.6%) had a PS of 3 to 4, indicating poor performance. Thoracic cancer was the most prevalent type of malignant neoplasm in this cohort, affecting 37 (24.8%) patients, followed by hepatobiliary tumors (cancer of the liver, biliary tract, and pancreas) in 34 (22.8%) and digestive tract cancer in 33 (22.1%). A real-world clinical study was conducted to compare the frequency of defecation before and 1 week after naldemedine treatment. The median frequencies of defecation in the entire population before and 7 days after naldemedine treatment were 3 (range, 0–15) and 6 (range, 0–49), respectively. Furthermore, the results demonstrated a significant increase in the frequency of defecation following naldemedine administration (Wilcoxon signed-rank test, *P < *.0001). In this study evaluating the efficacy and safety of naldemedine in patients with cancer with OIC in clinical practice, 65.7% of 149 patients responded.^[57]^

Shimizu et al conducted a multicenter, prospective, observational study using consecutive patient data to elucidate the safety and effectiveness of naldemedine in managing OIC in real-world oncology and palliative care settings.^[52]^ A total of 204 patients were enrolled, and 184 completed the 7-day study period. Within 24 hours of the first naldemedine dose, 146 patients (71.6%, 95% CI: 65.4%–77.8%) experienced SBMs. The weekly SBM counts increased in 62.7% of the participants.

### 4.4. Safety

In the COMPOSE-4 trial, a significantly higher proportion of patients treated with naldemedine experienced TEAEs than those treated with placebo (44.3% [43 of 97 patients] vs 26.0% [25 of 96 patients]; *P = *.01). Of the 131 patients in the COMPOSE-5 trial, 105 (80.2%) experienced TEAEs. The most frequently reported TEAEs in both studies were gastrointestinal disorders, the most common of which was diarrhea (COMPOSE-4: naldemedine, 19.6% [19 of 97 patients] vs placebo, 7.3% [7 of 96 patients] and COMPOSE-5: naldemedine, 18.3% [24 of 131 patients]). However, most of these TEAEs were mild to moderate in severity, and only a small number of patients in COMPOSE-4 (9.3%) discontinued the study.^[50]^

In a long-term post-marketing study, 1177 patients were analyzed.^[51]^ The patients had a mean age of 69.0 years (standard deviation: 12.8), with 70.1% aged ≥ 65 years and 57.1% being male. The majority exhibited an ECOG-PS of 1 (30.2%), 2 (27.2%), or ≥ 3 (32.4%). In total, 383 patients (32.5%) continued the naldemedine treatment throughout the 12-week study period. The primary reason for discontinuation in the safety analysis cohort was disease progression preventing naldemedine administration (43.3%; n = 344), followed by cancer-related mortality (20.0%; n = 159), adverse events (7.6%; n = 60), opioid withdrawal (6.9%; n = 55), clinical improvement (5.5%; n = 44), insufficient response (2.4%; n = 19), and other reasons (14.2%; n = 113). Adverse events were reported in 756 patients (64.23%), predominantly manifesting as symptoms associated with cancer progression. Both the reporter and physician excluded any causal link to naldemedine. A total of 145 adverse events were documented in 133 patients (11.30%), with gastrointestinal issues accounting for the majority of events (n = 121; 10.28%). Among the 145 events recorded, 136 (93.8%) were classified as non-severe. Over half (55.2%) emerged within the first week of treatment, with resolution occurring within 1 week in 60.0% and within 2 weeks in 75.9% of cases.^[51]^

Adverse events that were deemed to be causally related to naldemedine treatment included diarrhea, which was the most common adverse event of all grades, occurring in 57 (38.2%) patients. Of these, 52 (88.1%) were of grades 1 to 2. In the assessments using the Common Terminology Criteria for Adverse Events v5.0, no patients experienced grade 4 or higher adverse events.^[57]^

A post hoc integrated subgroup analysis^[60]^ included 2 randomized trials to assess the baseline characteristics that may influence the efficacy and safety of naldemedine in patients with OIC and cancer. A total of 307 patients (naldemedine, n = 155; placebo, n = 152) were included in this analysis, with pooled proportions of SBM responders of 73.5% and 35.5% in the naldemedine and placebo groups, respectively. The proportion of SBM responders was significantly higher with naldemedine than with placebo (38.0%, 95% CI, 27.6%–48.4%, *P < *.0001). In all subgroups, naldemedine increased the proportion of SBM responders and patients with diarrhea compared with placebo. Naldemedine (0.2 mg) did not affect analgesic efficacy or withdrawal symptoms, even in patients with possible blood–brain barrier impairment.^[60,61]^ However, naldemedine is an amide derivative of naltrexone with structural modifications that limit its ability to cross the blood–brain barrier and prevent the central analgesic effects of opioids. It is characterized by its ability to act exclusively in the periphery; however, case reports of opioid withdrawal syndrome have recently emerged despite the absence of brain metastases, and further verification is required.^[62–66]^

Shimizu et al reported that the major adverse events included diarrhea and abdominal pain, which were detected in 17.6% and 5.4% of the patients, respectively. However, no severe adverse events, such as gastrointestinal perforation, bleeding, or death, were reported.^[52]^

### 4.5. Breakdown of the data by cancer type:

A sub-analysis of real-world clinical trials of naldemedine use in thoracic, gastrointestinal, and hepatobiliary tumors was performed.^[67–69]^ Diarrhea was the most prevalent adverse event in 40 patients with thoracic tumors of any grade, occurring in 11 (27.5%) patients. Of these, 9 (81.8%) patients had grade 1 or 2. None of the patients experienced adverse events of grade 4 or higher.^[67]^ A total of 33 patients were diagnosed with gastrointestinal tumors.^[68]^ Of these, 39% and 9% exhibited grade 1 and 2 diarrhea, respectively. No grade 3 or 4 adverse events were observed. Notably, no other gastrointestinal symptoms were observed in patients with grades 2 to 4 diarrhea. The most prevalent adverse event in 34 patients with hepatobiliary tumors was diarrhea, which occurred in 10 (29.4%) patients (of any grade).^[69]^ Of these, 9 (90.0%) patients reported grade 1 or 2 diarrhea. However, none of the patients experienced adverse events of grade 4 or higher. Based on the above, safety does not appear to be compromised by the cancer type.

#### 4.5.1. Older patients

OIC in older patients (age > 65 years) is not widely recognized, and naldemedine, an oral PAMORA, has not been tested for its effectiveness and safety in this population.^[70]^ Although comprising patients without cancer, a combined analysis of 3 phase-III trials (COMPOSE-1, COMPOSE-2, and COMPOSE-3) examined the safety and effectiveness of naldemedine in patients ≥ 65 years of age for up to 12 weeks.^[71]^ This report showed that the number of patients ≥ 65 years of age who experienced TEAEs when treated with naldemedine (45.9%) was similar to that of those in the placebo (51.6%) and overall naldemedine (47.1%) groups. Naldemedine-treated patients also had a higher proportion of responders than placebo-treated patients overall (50.1% vs 34.1%, *P < *.0001) and in patients ≥ 65 years of age (51.8% vs 37.6%). In patients ≥ 65 years of age with chronic non-cancer pain, the safety and efficacy results were consistent with those of the overall patient population and matched the results of a previous study.^[71]^

However, a few reports exist on the use of naldemedine in real-world clinical practice in older patients with cancer. Older patients in the phase-III trial were of ≥ 65 years of age, whereas in another report, they were of ≥ 75 years of age. Imai et al report examined the efficacy of naldemedine in treating OIC in 60 patients with cancer of ≥ 75 years of age.^[72]^ The response rate was 68.3%, and defecation frequency significantly increased after naldemedine treatment (*P < *.0001) in the overall population and in those whose frequency was < 3 times per week before treatment (*P* < .0001). Additionally, the increase in bowel movements was mostly due to diarrhea, which was the most common side effect across all grades and occurred in 45% of the patients, 92.6% of whom experienced grade 1 or 2 symptoms. No instances of adverse events of grade 4 or higher, including death, were observed in this patient cohort.^[72]^ Therefore, the results of this study indicate that naldemedine is not of significant concern in older adults.

#### 4.5.2. Pediatric patients

Studies on the use of PAMORAs for OIC in children are limited.^[73–77]^ Although no studies have assessed naldemedine use in pediatric patients with cancer, the response rate, defined as defecation within 4 hours, was 52%; the incidence of side effects using the Naranjo score revealed abdominal pain in 25% and nausea in 23% in a retrospective study evaluating patients < 18 years of age who received a single dose (0.15 mg/kg) of methylnaltrexone.^[73]^ Miura et al recently studied the efficacy and safety of naldemedine in pediatric patients.^[78]^ The patients were < 19 years of age and received naldemedine (median age, 9 [range 0.6–18] years). The median opioid dose was 0.5 (range 6–11.7) mg/kg/d and 0.7 (range 6–10.5) mg/kg/d for 7 days before and after naldemedine use, respectively. Two patients received naldemedine simultaneously with the initiation of opioid therapy. The median dose of naldemedine was 0.0058 (range 0.0012–0.020) mg/kg/d. The evaluation method was based on patients who had defecated at least 3 times a week after naldemedine administration and responders were those who had increased defecation frequency at least once within 7 days after administration. In addition to the number of bowel movements before and after naldemedine administration, additional laxative use within 7 days of initiation was also evaluated. Safety was evaluated based on adverse events (Common Terminology Criteria for Adverse Events, v.5.0) that occurred within 7 days of naldemedine administration. Eleven of 13 (85%) patients had defecation. The median frequency of defecation ranged from 0.43 to 1.0 per day. Diarrhea was present in 16 of 32 patients (50%). The dose of naldemedine in the 4 patients with grade-2 diarrhea was 0.0075 (range 0.0070–0.0086) mg/kg/d, and none had grade 3. The dose of naldemedine for the remaining 28 patients was 0.0058 (range 0.0012–0.020) mg/kg/d. Three other patients had abdominal pain, but no opioid withdrawal symptoms such as vomiting or sweating were reported. In this study, grade-2 diarrhea was reported with the lowest dose of 0.0070 mg/kg/d, suggesting that it may have been equivalent to the higher adult dose of 0.4 mg/dose. This study was small and showed efficacy, but it also indicated caution in pediatric patients, as grade-2 diarrhea resulted in discontinuation of the drug.

#### 4.5.3. Patients with poor ECOG-PS

An ECOG-PS of 3 to 4 was an exclusion criterion in the naldemedine randomized phase-III trial^[50]^; however, post-marketing surveillance included ECOG-PS failure. In a report by Naya et al, 29.4% and 37.2% of patients ≥ 75 years and < 75 years of age, respectively, had an ECOG-PS of 3 to 4. The TEAEs in patients with ECOG-PS 3 to 4 were significantly lower than those in individuals with ECOG-PS 0 to 2 (8.40% vs 12.70%, *P = *.0291). This report showed that the number of patients who experienced side effects while undergoing concurrent cancer treatment was higher than that of those who did not undergo cancer treatment (15.58% vs 8.84%, *P = *.0004 for overall side effects; 13.02% vs 6.83%, *P = *.0004 for diarrhea).^[59]^ Fujita et al obtained the results of real-life clinical studies. The objective of their study was to assess the efficacy and safety of naldemedine in patients with cancer and poor ECOG-PS who were receiving opioids.^[54]^ The number of bowel movements before and after naldemedine administration was analyzed. Responders were defined as patients whose defecation frequency increased to ≥ 3 times/week, from a baseline of ≤ 1 defecation per week, over 7 days after naldemedine administration. The study analyzed data from 71 patients, of whom 66.1% were responders (95% CI, 54.5%–76.1%). The frequency of defecation significantly increased after naldemedine administration in both the overall population (6 times/week vs 2 times/week*, P < *.0001) and among those who defecated < 3 times/week before naldemedine administration (4.5 times/week vs 1 time/week, *P < *.0001). Specifically, the most common adverse event was diarrhea (38.0% of all cases), with 23 (85.2%) events being classified as grade 1 or 2. Naldemedine was found to be effective and safe among patients with cancer and poor ECOG-PS.^[54]^ Additionally, naldemedine has demonstrated efficacy in patients with cancer cachexia. Nakatsugawa et al revealed that the number of patients who responded with SBMs increased when naldemedine treatment was initiated within 2 days of opioid treatment.^[79]^

## 5. Concurrent or early opioid administration

OIC is treated with conventional constipation medications such as osmotic laxatives; however, naldemedine is recommended if no improvement occurs.^[80]^ Japanese guidelines recommend PAMORAs for refractory OIC without opioid switching, conventional colorectal stimulation, or osmotic laxative improvement.^[81]^ However, in practice, naldemedine is administered early after opioid administration in some cases. In these cases, the most common side effect, diarrhea, was milder than that after prolonged opioid administration.^[55,56]^ A study compared the effectiveness of magnesium oxide and naldemedine in the prophylactic treatment of OIC and showed that naldemedine was more effective.^[53]^ However, Yamada et al reported that naldemedine alone was ineffective against OIC.^[82]^ In their study, they demonstrated the poor prophylactic effect of naldemedine on OIC, along with the factors influencing this effect. In patients who started naldemedine for the prevention of OIC at the time of introduction of oxycodone tablets, a significant worsening was observed in the Support Team Assessment Schedule Japanese edition score after the introduction of oxycodone tablets, which was evaluated by medical personnel, as well as in the frequency of defecation, Bristol stool form scale score, and development or worsening of straining to pass bowel movements, which are patient-reported outcomes. These results suggest that naldemedine alone does not prevent OIC induced by oxycodone. Hamano et al presented the results of a clinical trial designed to confirm the prophylactic effect of naldemedine on OIC in patients with cancer with initiation of daily administration of strong opioids.^[37]^ Eligible patients were randomly assigned to the naldemedine or placebo group on a 1:1 basis. The protocol treatment period was 14 days after the initiation of naldemedine (or placebo) administration, and the naldemedine group was administered 0.2 mg of naldemedine once daily after breakfast for 14 days. The primary endpoint was the proportion of patients with a bowel function indexbowel function index (BFI) of < 28.8 on day 14. The proportion of patients with a bowel function index < 28.8 on day 14 was significantly greater with naldemedine (64.6% [31 of 48 patients]; 95% CI, 51.1%–78.1%) than with placebo (17.0% [8 of 47 patients]; 95% CI, 6.3%–27.8%), with a difference of 47.6% (95% CI, 30.3%–64.8%, *P < *.0001). No significant difference was found in the proportion of adverse events, such as abdominal distention, abdominal pain, diarrhea, bowel obstruction, or nausea. However, a significantly lower proportion of vomiting was observed in patients treated with naldemedine. The possibility of reduced nausea during naldemedine administration has been shown in animal studies and clinical practice.^[38,83]^ Naldemedine is a valuable option with proven efficacy in preventing OIC in patients with cancer who are starting regular administration of strong opioids.^[37]^ Therefore, accumulating data based on clinical practice in the future is necessary. Moreover, if the number of cases continues to increase, concurrent administration of opioids may be feasible.

## 6. Efficacy and safety of naldemedine for OIC caused by weak opioids

Weak opioids are widely used to treat cancer pain, but the information on the efficacy and safety of naldemedine for OIC caused by weak opioids is limited. The results of a subgroup analysis of a post-marketing survey by Naya et al showed no difference in the efficacy of naldemedine in improving the frequency and condition of bowel movements regardless of the type of opioid analgesic used, whether strong or weak opioids.^[59]^ Similarly, a post-marketing survey by Takata et al also found that the effect was the same regardless of the strength of the opioid. Furthermore, no statistically significant difference was reported in the incidence of diarrhea, the most common adverse event, between weak and strong opioids.^[51]^ The mechanism of this effect has been reported in a basic experiment in rodents, which involved the peripheral μ-opioid receptors in tramadol-induced constipation. The mechanism is supported by the fact that peripheral μ-opioid receptors may also be involved in tramadol-induced constipation.^[84]^

## 7. Efficacy and side effects when co-administered with other laxatives

Naldemedine is often used in combination with other laxatives in clinical practice, thus making it important to consider its efficacy and side effects in combination with other laxatives. In a post-marketing survey by Takata et al, 72.6% (n = 854) of patients had used laxatives prior to initiating naldemedine treatment, with 90.9% (n = 776) of these individuals continuing their laxative regimen thereafter. The majority (80.7%; n = 950) used concomitant laxatives, predominantly osmotic/saline laxatives (63.2%; n = 744) and/or stimulant/colorectal-stimulant laxatives (33.6%; n = 396). No statistically significant difference was reported in the incidence of diarrhea with or without laxative combination, which was the most commonly observed adverse event.^[51]^ In addition, subgroup analysis of the pooled analysis of the 2 randomized clinical trials of naldemedine by Osaka et al showed that the response rate of SBM and the incidence of diarrhea in the subgroup with or without the use of laxatives were generally consistent with those observed in the overall pooled patient population.^[60]^ These data suggest that the use or nonuse of laxatives did not affect the efficacy or safety of naldemedine at 0.2 mg in patients with OIC and cancer. Furthermore, the pooled post hoc subgroup analysis reported by Kessou et al was conducted using the data from patients who participated in the previous phase-IIb and -III clinical trials of naldemedine targeting patients with cancer and OIC, who had been taking magnesium oxide before starting naldemedine and continued its use during naldemedine treatment.^[85]^ According to the report, the naldemedine group showed significant improvements (*P < *.0001) in the frequency of SBM and complete SBM, rate of improvement of SBM, and rate of improvement of complete SBM compared to the placebo group. These results suggest that the effects of naldemedine, which has a different mechanism of action than that of magnesium oxide, are independent of magnesium oxide. Furthermore, in this report, although the incidence of adverse events and diarrhea was significantly higher (*P < *.05) in the naldemedine group than in the placebo group, no significant difference was reported between the 2 groups in the incidence of serious adverse events, death, adverse events leading to discontinuation of treatment, or diarrhea. This suggests that the addition of naldemedine to continuous treatment with magnesium oxide does not raise any new safety concerns. The study demonstrated that the addition of naldemedine to continuous treatment with magnesium oxide for OIC in patients with cancer is both effective and well-tolerated.

## 8. Future possibilities excluding OIC

The future potential of naldemedine in relation to its effects on opioid-induced nausea and vomiting,^[83]^ delirium, and tumors have been reported. The use of naldemedine from the beginning of opioid administration in patients with constipation has been suggested to have the potential to bring secondary benefits such as the alleviation of opioid-induced nausea and vomiting in addition to the improvement of OIC.^[83]^ In addition, the use of naldemedine in patients with cancer undergoing chemotherapy and opioid therapy has been suggested to have the potential to be beneficial in preventing agitation delirium, shortening the duration of hospital stay, and reducing hospitalization costs.^[86]^ Furthermore, although the study was conducted using mice, the findings suggested that blocking peripheral μ-receptors by administering naldemedine may have an antitumor effect by restoring T-cell exhaustion and promoting the tumor-killing system.^[87]^ The above-mentioned blocking of peripheral μ-opioid receptors by naldemedine may facilitate the exploration of the potential of naldemedine in the future.

## 9. Conclusion

Most previous reviews focused on the results of major prospective trials and did not consider differences such as cancer type, older patients, children, ECOG-PS, or combination therapy; however, our review differs from these reports by including reviews of real-world data and post-marketing clinical trials.

This review underscores the clinical importance of managing OIC in patients with cancer, highlighting naldemedine use as an effective and well-tolerated treatment. The findings indicate that naldemedine alleviates OIC without compromising opioid analgesia or causing withdrawal symptoms. Effective OIC management is crucial for maintaining opioid adherence and improving patients’ quality of life.

OIC is the most common adverse effect of opioids, and nonspecific interventions are ineffective in its treatment. PAMORAs are a class of treatments based on the mechanism of OIC, and methylnaltrexone, naloxegol, and naldemedine are the 3 currently available PAMORAs. This narrative review focuses on naldemedine and presents data on its efficacy and safety in patients with cancer, along with recent reports. The efficacy and safety profiles of naldemedine were comparable to those in patients without cancer. Furthermore, naldemedine was well-tolerated by patients with poor ECOG-PS and possible brain metastases. Naldemedine can prevent OIC with early administration and may also prevent nausea. Therefore, naldemedine is a safe, effective, and well-tolerated drug for the treatment of OIC in patients with cancer. However, most of the research targets Japanese patients, and the findings may not be generalizable to patients from other countries.

Future research should focus on the long-term safety and efficacy of naldemedine, its role in combination therapies, and its impact on different cancer subtypes. Real-world data will further clarify its benefits, helping to optimize OIC treatment strategies and enhance supportive care for patients with cancer.

## Acknowledgments

We would like to thank Editage (www.editage.jp) for English language editing.

## Author contributions

**Conceptualization:** Hisao Imai.

**Funding acquisition:** Kyoko Obayashi.

**Methodology:** Hiromi Nishiba, Hisao Imai.

**Software:** Hiromi Nishiba.

**Supervision:** Kyoko Obayashi.

**Validation:** Hisao Imai.

**Visualization:** Hiromi Nishiba, Hisao Imai.

**Writing – original draft:** Hiromi Nishiba, Hisao Imai.

**Writing – review & editing:** Ayumu Nagamine, Yuta Takahashi, Kyoko Obayashi.

## References

[1] Bouhassira D Luporsi E Krakowski I. Prevalence and incidence of chronic pain with or without neuropathic characteristics in patients with cancer. Pain. 2017;158:1118–25. 28267066 10.1097/j.pain.0000000000000895

[2] Snijders RAH Brom L Theunissen M van den Beuken-van Everdingen MHJ. Update on prevalence of pain in patients with cancer 2022: a systematic literature review and meta-analysis. Cancers (Basel). 2023;15:591. 36765547 10.3390/cancers15030591 PMC9913127

[3] Tavoli A Montazeri A Roshan R Tavoli Z Melyani M. Depression and quality of life in cancer patients with and without pain: the role of pain beliefs. BMC Cancer. 2008;8:177. 18570676 10.1186/1471-2407-8-177 PMC2443809

[4] Khan F Mehan A. Addressing opioid tolerance and opioid-induced hypersensitivity: recent developments and future therapeutic strategies. Pharmacol Res Perspect. 2021;9:e00789. 34096178 10.1002/prp2.789 PMC8181203

[5] Benyamin R Trescot AM Datta S. Opioid complications and side effects. Pain Physician. 2008;11:S105–20. 18443635

[6] Hooten WM Lamer TJ Twyner C. Opioid-induced hyperalgesia in community-dwelling adults with chronic pain. Pain. 2015;156:1145–52. 25815431 10.1097/j.pain.0000000000000170 PMC4431900

[7] Coyne KS Margolis MK Yeomans K. Opioid-induced constipation among patients with chronic noncancer pain in the United States, Canada, Germany, and the United Kingdom: laxative use, response, and symptom burden over time. Pain Med. 2015;16:1551–65. 25802051 10.1111/pme.12724

[8] Coyne KS LoCasale RJ Datto CJ Sexton CC Yeomans K Tack J. Opioid-induced constipation in patients with chronic noncancer pain in the USA, Canada, Germany, and the UK: descriptive analysis of baseline patient-reported outcomes and retrospective chart review. Clinicoecon Outcomes Res. 2014;6:269–81. 24904217 10.2147/CEOR.S61602 PMC4041290

[9] Mearin F Lacy BE Chang L. Bowel disorders. Gastroenterology. 2016;16;S0016-5085(16)00222-5. 10.1053/j.gastro.2016.02.031 27144627

[10] Davis MP. Cancer constipation: are opioids really the culprit? Support Care Cancer. 2008;16:427–9. 18196285 10.1007/s00520-007-0386-9

[11] Davies A Leach C Butler C. Opioid-induced constipation in patients with cancer: a “real-world,” multicentre, observational study of diagnostic criteria and clinical features. Pain. 2021;162:309–18. 32701649 10.1097/j.pain.0000000000002024

[12] Tokoro A Imai H Fumita S. Incidence of opioid-induced constipation in Japanese patients with cancer pain: a prospective observational cohort study. Cancer Med. 2019;8:4883–91. 31231974 10.1002/cam4.2341 PMC6712473

[13] Lang-Illievich K Bornemann-Cimenti H. Opioid-induced constipation: a narrative review of therapeutic options in clinical management. Korean J Pain. 2019;32:69–78. 31091505 10.3344/kjp.2019.32.2.69 PMC6549585

[14] Staats PS Markowitz J Schein J. Incidence of constipation associated with long-acting opioid therapy: a comparative study. South Med J. 2004;97:129–34. 14982259 10.1097/01.SMJ.0000109215.54052.D8

[15] Li Y Ma J Lu G. Hydromorphone for cancer pain. Cochrane Database Syst Rev. 2021;8:CD011108. 34350974 10.1002/14651858.CD011108.pub3 PMC8406835

[16] Brock C Olesen SS Olesen AE Frøkjaer JB Andresen T Drewes AM. Opioid-induced bowel dysfunction: pathophysiology and management. Drugs. 2012;72:1847–65. 22950533 10.2165/11634970-000000000-00000

[17] Farmer AD Drewes AM Chiarioni G. Pathophysiology and management of opioid-induced constipation: European expert consensus statement. United Eur Gastroenterol J. 2019;7:7–20. 10.1177/2050640618818305 PMC6374852 30788113

[18] Sternini C Patierno S Selmer IS Kirchgessner A. The opioid system in the gastrointestinal tract. Neurogastroenterol Motil. 2004;16:3–16. 10.1111/j.1743-3150.2004.00553.x 15357847

[19] Zöllner C Stein C. Opioids. Handb Exp Pharmacol. 2007;177:31–63. 10.1007/978-3-540-33823-9_2 17087119

[20] Sharma SK Nirenberg M Klee WA. Morphine receptors as regulators of adenylate cyclase activity. Proc Natl Acad Sci U S A. 1975;72:590–4. 1054841 10.1073/pnas.72.2.590 PMC432359

[21] Wood JD Galligan JJ. Function of opioids in the enteric nervous system. Neurogastroenterol Motil. 2004;16:17–28. 15357848 10.1111/j.1743-3150.2004.00554.x

[22] Argoff CE. Opioid-induced constipation: a review of health-related quality of life, patient burden, practical clinical considerations, and the impact of peripherally acting μ-opioid receptor antagonists. Clin J Pain. 2020;36:716–22. 32554978 10.1097/AJP.0000000000000852 PMC7473817

[23] Sykes NP. The relationship between opioid use and laxative use in terminally ill cancer patients. Palliat Med. 1998;12:375–82. 9924600 10.1191/026921698674125048

[24] Bell TJ Panchal SJ Miaskowski C Bolge SC Milanova T Williamson R. The prevalence, severity, and impact of opioid-induced bowel dysfunction: results of a US and European Patient Survey (PROBE 1). Pain Med. 2009;10:35–42. 18721170 10.1111/j.1526-4637.2008.00495.x

[25] Fine PG Chen YW Wittbrodt E Datto C. Impact of opioid-induced constipation on healthcare resource utilization and costs for cancer pain patients receiving continuous opioid therapy. Support Care Cancer. 2019;27:687–96. 30056531 10.1007/s00520-018-4366-z

[26] Poulsen JL Brock C Olesen AE Nilsson M Drewes AM. Evolving paradigms in the treatment of opioid-induced bowel dysfunction. Therap Adv Gastroenterol. 2015;8:360–72. 10.1177/1756283X15589526 PMC4622283 26557892

[27] Cook SF Lanza L Zhou X. Gastrointestinal side effects in chronic opioid users: results from a population-based survey. Aliment Pharmacol Ther. 2008;27:1224–32. 18363893 10.1111/j.1365-2036.2008.03689.x

[28] Wirz S Nadstawek J Elsen C Junker U Wartenberg HC. Laxative management in ambulatory cancer patients on opioid therapy: a prospective, open-label investigation of polyethylene glycol, sodium picosulphate and lactulose. Eur J Cancer Care (Engl). 2012;21:131–40. 21880080 10.1111/j.1365-2354.2011.01286.x

[29] Lemaire A Pointreau Y Narciso B Piloquet FX Braniste V Sabaté JM. Effectiveness of naloxegol in patients with cancer pain suffering from opioid-induced constipation. Support Care Cancer. 2021;29:7577–86. 34120247 10.1007/s00520-021-06299-2 PMC8549935

[30] Webster LR Israel RJ. Oral methylnaltrexone does not negatively impact analgesia in patients with opioid-induced constipation and chronic noncancer pain. J Pain Res. 2018;11:1503–10. 30147355 10.2147/JPR.S160488 PMC6095122

[31] Braun UK Jackson LK Garcia MA Imam SN. A systematic review of naldemedine and naloxegol for the treatment of opioid-induced constipation in cancer patients. Pharmacy (Basel). 2024;12:48. 38525728 10.3390/pharmacy12020048 PMC10961755

[32] Blair HA. Naldemedine: a review in opioid-induced constipation. Drugs. 2019;79:1241–7. 31267482 10.1007/s40265-019-01160-7

[33] Vijayvargiya P Camilleri M Vijayvargiya P Erwin P Murad MH. Systematic review with meta-analysis: efficacy and safety of treatments for opioid-induced constipation. Aliment Pharmacol Ther. 2020;52:37–53. 32462777 10.1111/apt.15791

[34] Rekatsina M Paladini A Drewes AM. Efficacy and safety of peripherally acting μ-opioid receptor antagonist (PAMORAs) for the management of patients with opioid-induced constipation: a systematic review. Cureus. 2021;13:e16201. 34367804 10.7759/cureus.16201 PMC8339109

[35] Pergolizzi JV Jr. Christo PJ LeQuang JA Magnusson P. The use of peripheral μ-opioid receptor antagonists (PAMORA) in the management of opioid-induced constipation: an update on their efficacy and safety. Drug Des Devel Ther. 2020;14:1009–25. 10.2147/DDDT.S221278 PMC7075239 32210534

[36] Tack J Lappalainen J Diva U Tummala R Sostek M. Efficacy and safety of naloxegol in patients with opioid-induced constipation and laxative-inadequate response. United Eur Gastroenterol J. 2015;3:471–80. 10.1177/2050640615604543 PMC4625753 26535126

[37] Hamano J Higashibata T Kessoku T. Naldemedine for opioid-induced constipation in patients with cancer: a multicenter, double-blind, randomized, placebo-controlled trial. J Clin Oncol. 2024;42:4206–17. 39255425 10.1200/JCO.24.00381 PMC11637578

[38] Kanemasa T Matsuzaki T Koike K Hasegawa M Suzuki T. Preventive effects of naldemedine, peripherally acting μ-opioid receptor antagonist, on morphine-induced nausea and vomiting in ferrets. Life Sci. 2020;257:118048. 32622946 10.1016/j.lfs.2020.118048

[39] Webster LR Yamada T Arjona Ferreira JC. A phase 2b, randomized, double-blind placebo-controlled study to evaluate the efficacy and safety of naldemedine for the treatment of opioid-induced constipation in patients with chronic noncancer pain. Pain Med. 2017;18:2350–60. 28371937 10.1093/pm/pnw325 PMC5914456

[40] FDA. Drug Approvals and Databases; Symproic (naldemedine) Tablets. Available at: https://www.accessdata.fda.gov/drugsatfda_docs/nda/2017/208854Orig1s000TOC.cfm [Accessed April 7, 2024]

[41] Kanemasa T Koike K Takase K. Pharmacological profile of naldemedine, a peripherally acting μ-opioid receptor antagonist: comparison with naloxone and naloxegol. J Pharmacol Exp Ther. 2020;373:438–44. 32169839 10.1124/jpet.119.264515

[42] Watari R Matsuda A Ohnishi S Hasegawa H. Minimal contribution of P-gp on the low brain distribution of naldemedine, a peripherally acting μ-opioid receptor antagonist. Drug Metab Pharmacokinet. 2019;34:126–33. 30770183 10.1016/j.dmpk.2018.12.002

[43] Galligan JJ Akbarali HI. Molecular physiology of enteric opioid receptors. Am J Gastroenterol Suppl. 2014;2:17–21. 25207608 10.1038/ajgsup.2014.5 PMC4426191

[44] Kanemasa T Koike K Arai T. Pharmacologic effects of naldemedine, a peripherally acting μ-opioid receptor antagonist, in in vitro and in vivo models of opioid-induced constipation. Neurogastroenterol Motil. 2019;31:e13563. 30821019 10.1111/nmo.13563 PMC6850587

[45] BouSaba J Sannaa W Camilleri M. Update on the role of naldemedine in opioid-induced constipation in patients with chronic noncancer pain. Therap Adv Gastroenterol. 2022;15:17562848221078638. 10.1177/17562848221078638 PMC9058332 35509419

[46] Fukumura K Yamada T Yokota T Kawasaki A. The influence of renal or hepatic impairment on the pharmacokinetics, safety, and tolerability of naldemedine. Clin Pharmacol Drug Dev. 2020;9:162–74. 30977959 10.1002/cpdd.690 PMC7027783

[47] Fukumura K Kawaguchi N Ishibashi T Kubota R Tada Y Ogura E. Clinical drug-drug interaction studies to evaluate the effects of a p-glycoprotein inhibitor, CYP3A inhibitors, and a CYP3A inducer on the pharmacokinetics of naldemedine in healthy subjects. Clin Drug Investig. 2020;40:529–40. 10.1007/s40261-020-00902-w PMC7242238 32323104

[48] Raffa RB Taylor R Jr. Pergolizzi JV Jr. Treating opioid-induced constipation in patients taking other medications: avoiding CYP450 drug interactions. J Clin Pharm Ther. 2019;44:361–71. 30793348 10.1111/jcpt.12812

[49] Katakami N Oda K Tauchi K. Phase IIb, randomized, double-blind, placebo-controlled study of naldemedine for the treatment of opioid-induced constipation in patients with cancer. J Clin Oncol. 2017;35:1921–8. 28445097 10.1200/JCO.2016.70.8453

[50] Katakami N Harada T Murata T. Randomized phase III and extension studies of naldemedine in patients with opioid-induced constipation and cancer. J Clin Oncol. 2017;35:3859–66. 28968171 10.1200/JCO.2017.73.0853

[51] Takata K Nakazawa M Honda K Hashimoto S. Post-marketing surveillance of the safety and effectiveness of naldemedine in the management of opioid-induced constipation in patients with cancer pain in Japan. Support Care Cancer. 2022;30:3943–54. 35044484 10.1007/s00520-022-06807-y PMC8942924

[52] Shimizu M Maeda I Kessoku T; Phase-R OIC Study Group. The safety and effectiveness of naldemedine for opioid-induced constipation in patients with advanced cancer in real-world palliative care settings: a multicenter prospective observational study. Support Care Cancer. 2024;32:504. 38985364 10.1007/s00520-024-08716-8

[53] Ozaki A Kessoku T Tanaka K. Effectiveness of naldemedine compared with magnesium oxide in preventing opioid-induced constipation: a randomized controlled trial. Cancers (Basel). 2022;14:2112. 35565243 10.3390/cancers14092112 PMC9102438

[54] Fujita Y Imai H Hiruta E. Efficacy and safety of naldemedine administration for opioid-induced constipation in cancer patients with poor performance status. J Palliat Med. 2023;26:548–53. 36971576 10.1089/jpm.2022.0495

[55] Takagi Y Osawa G Kato Y Ikezawa E Kobayashi C Aruga E. Prevention and management of diarrhea associated with naldemedine among patients receiving opioids: a retrospective cohort study. BMC Gastroenterol. 2020;20:25. 32005157 10.1186/s12876-020-1173-z PMC6995158

[56] Hiruta E Fujita Y Imai H. Real-world patient characteristics and treatment patterns of naldemedine for the treatment of opioid-induced constipation in patients with cancer: a multicenter retrospective chart review study. Medicina (Kaunas). 2021;57:1233. 34833451 10.3390/medicina57111233 PMC8625056

[57] Nishiba H Imai H Fujita Y. Efficacy and safety of naldemedine for patients with cancer with opioid-induced constipation in clinical practice: a real-world retrospective study. J Clin Med. 2022;11:2672. 35566798 10.3390/jcm11092672 PMC9102706

[58] Fukumura K Yokota T Baba Y Arjona Ferreira JC. Phase 1, randomized, double-blind, placebo-controlled studies on the safety, tolerability, and pharmacokinetics of naldemedine in healthy volunteers. Clin Pharmacol Drug Dev. 2018;7:474–83. 28960888 10.1002/cpdd.387

[59] Naya N Oka H Hashimoto S Morioka Y Kizawa Y. Real- world evidence for the safety and effectiveness of naldemedine in the management of opioid-induced constipation in patients with cancer pain: post-hoc subgroup analysis of post-marketing surveillance in Japan. Cureus. 2023;15:e46090. 37900431 10.7759/cureus.46090 PMC10611588

[60] Osaka I Ishiki H Yokota T. Safety and efficacy of naldemedine in cancer patients with opioid-induced constipation: a pooled, subgroup analysis of two randomised controlled studies. ESMO Open. 2019;4:e000527. 31423335 10.1136/esmoopen-2019-000527 PMC6677965

[61] Hanamoto A Koseki T Utsunomiya A. Influence of brain metastasis on analgesia-related outcomes in patients with lung and breast cancers treated with naldemedine: a propensity score-matched analysis. J Clin Med. 2023;12:6997. 38002612 10.3390/jcm12226997 PMC10672656

[62] Ishida M Uchida N Yabuno A. Opioid withdrawal syndrome developing after long-term administration of naldemedine. Palliat Support Care. 2022;20:897–9. 35543119 10.1017/S147895152200044X

[63] Ishida M Hiraoka M Yaguchi A. Naldemedine-induced opioid withdrawal syndrome with severe psychiatric symptoms in an advanced cervical cancer patient without brain metastasis. Palliat Support Care. 2022;20:445–7. 34955117 10.1017/S1478951521001917

[64] Ishii K Yamashita H Yamaguchi M. Naldemedine-induced opioid withdrawal syndrome in a patient with breast cancer without brain metastasis. Intern Med. 2020;59:293–6. 31534081 10.2169/internalmedicine.3098-19 PMC7008055

[65] Sato R Ishida M Uchida N. Naldemedine-induced opioid withdrawal with restlessness as the predominant symptom in a palliative care setting. Palliat Support Care. 2023;21:957–9. 37350233 10.1017/S1478951523000858

[66] Candy B Jones L Vickerstaff V Larkin PJ Stone P. Mu-opioid antagonists for opioid-induced bowel dysfunction in people with cancer and people receiving palliative care. Cochrane Database Syst Rev. 2018;6:CD006332. 29869799 10.1002/14651858.CD006332.pub3 PMC6513061

[67] Imai H Fujita Y Hiruta E. A retrospective study of the efficacy and safety of naldemedine for opioid-induced constipation in thoracic cancer patients. Thorac Cancer. 2022;13:2301–8. 35790500 10.1111/1759-7714.14557 PMC9376157

[68] Nishiba H Imai H Fujita Y. Efficacy and safety of naldemedine treatment for opioid-induced constipation in gastrointestinal cancer: a retrospective analysis. Ann Palliat Med. 2023;12:697–707. 37081703 10.21037/apm-22-1130

[69] Kamiya T Imai H Fujita Y. A retrospective study of the efficacy and safety of naldemedine for treatment of opioid-induced constipation in patients with hepatobiliary pancreatic cancer. Medicina (Kaunas). 2023;59:492. 36984494 10.3390/medicina59030492 PMC10051263

[70] Chokhavatia S John ES Bridgeman MB Dixit D. Constipation in elderly patients with noncancer pain: focus on opioid-induced constipation. Drugs Aging. 2016;33:557–74. 27417446 10.1007/s40266-016-0381-2 PMC5012150

[71] Wild J Webster L Yamada T Hale M. Safety and efficacy of naldemedine for the treatment of opioid-induced constipation in patients with chronic non-cancer pain receiving opioid therapy: a subgroup analysis of patients ≥ 65 years of age. Drugs Aging. 2020;37:271–9. 32086791 10.1007/s40266-020-00753-2 PMC7096364

[72] Imai H Fujita Y Hiruta E. Efficacy and safety of naldemedine for opioid-induced constipation in older patients with cancer: a retrospective study. Eur J Gastroenterol Hepatol. 2024;36:571–7. 38477855 10.1097/MEG.0000000000002746

[73] Raschka M Gahr K Watson D Lu M. Clinical outcomes of intravenous methylnaltrexone in children: a single-arm retrospective cohort study. J Pediatr Pharmacol Ther. 2024;29:292–8. 38863861 10.5863/1551-6776-29.3.292 PMC11163904

[74] Mills KP McPherson CC Said AS Lahart MA. Successful use of intravenous methylnaltrexone for opioid-induced constipation in critically ill pediatric patients. J Pediatr Intensive Care. 2024;13:25–31. 38571990 10.1055/s-0041-1736335 PMC10987215

[75] Smith CJ Sierra CM Robbins J Chang NY Mirza F. Methylnaltrexone for opioid-induced dysmotility in critically ill infants and children: a pilot study. J Pediatr Pharmacol Ther. 2023;28:136–42. 37139255 10.5863/1551-6776-28.2.136 PMC10150904

[76] Rodrigues A Wong C Mattiussi A Alexander S Lau E Dupuis LL. Methylnaltrexone for opioid-induced constipation in pediatric oncology patients. Pediatr Blood Cancer. 2013;60:1667–70. 23766091 10.1002/pbc.24615

[77] Gillett EL Layes CA Crawley L Schexnayder SM. Naloxegol for treatment of opioid-induced constipation in the pediatric intensive care unit. Clin Pediatr (Phila). 2023;62:721–4. 36475875 10.1177/00099228221142129

[78] Miura R Utano T Miura Y. Efficacy safety of naldemedine for opioid-induced constipation in children. J Palliat Med. 2024;27:1354–8. 39122251 10.1089/jpm.2024.0018

[79] Nakatsugawa E Naito T Shibata K Kitajima R Kawakami J. Impacts of genetic polymorphisms and cancer cachexia on naldemedine pharmacokinetics and bowel movements in patients receiving opioid analgesics. Fundam Clin Pharmacol. 2024;38:596–605. 38192190 10.1111/fcp.12976

[80] Crockett SD Greer KB Heidelbaugh JJ Falck-Ytter Y Hanson BJ Sultan S; American Gastroenterological Association Institute Clinical Guidelines Committee. American Gastroenterological Association Institute Guideline on the medical management of opioid-induced constipation. Gastroenterology. 2019;156:218–26. 30340754 10.1053/j.gastro.2018.07.016

[81] Mawatari H Shinjo T Morita T Kohara H Yomiya K. Revision of pharmacological treatment recommendations for cancer pain: clinical guidelines from the Japanese society of palliative medicine. J Palliat Med. 2022;25:1095–114. 35363057 10.1089/jpm.2021.0438

[82] Yamada M Jimaru Y Torii S Mitsuba N Takahashi K. A retrospective observational study of factors affecting the efficacy of concurrent prescription of naldemedine for opioid-induced constipation caused by oxycodone tablets. Biol Pharm Bull. 2023;46:1826–31. 38044102 10.1248/bpb.b23-00487

[83] Sato J Tanaka R Ishikawa H Suzuki T Shino M. A preliminary study of the effect of naldemedine tosylate on opioid-induced nausea and vomiting. Support Care Cancer. 2020;28:1083–8. 31183560 10.1007/s00520-019-04884-0

[84] Yasufuku K Koike K Kobayashi M. Involvement of the peripheral μ-opioid receptor in tramadol-induced constipation in rodents. Biol Pharm Bull. 2021;44:1746–51. 34719650 10.1248/bpb.b21-00474

[85] Kessoku T Akamatsu T Morioka Y. Effect of add-on naldemedine treatment in patients with cancer and opioid-induced constipation insufficiently responding to magnesium oxide: a pooled, subgroup analysis of two randomized controlled trials. Jpn J Clin Oncol. 2025;55:40–8. 39354673 10.1093/jjco/hyae135 PMC11708229

[86] Abe H Sumitani M Matsui H. Use of naldemedine is associated with reduced incidence of hyperactive delirium in cancer patients with opioid-induced constipation: a nationwide retrospective cohort study in Japan. Pharmacotherapy. 2022;42:241–9. 34967450 10.1002/phar.2658

[87] Gondoh E Hamada Y Mori T. Possible mechanism for improving the endogenous immune system through the blockade of peripheral μ-opioid receptors by treatment with naldemedine. Br J Cancer. 2022;127:1565–74. 35945243 10.1038/s41416-022-01928-x PMC9553910

